# Stereophysicochemical variability plots highlight conserved antigenic areas in Flaviviruses

**DOI:** 10.1186/1743-422X-2-40

**Published:** 2005-04-21

**Authors:** Catherine H Schein, Bin Zhou, Werner Braun

**Affiliations:** 1 Sealy Center for Structural Biology, Department of Human Biology, Chemistry and Genetics, University of Texas Medical Branch at Galveston, TX, USA; 2 Sealy Center for Vaccine Development, Department of Human Biology, Chemistry and Genetics, University of Texas Medical Branch at Galveston, TX, USA; 3 Department of Microbiology and Immunology, University of Texas Medical Branch at Galveston, TX, USA

## Abstract

**Background:**

Flaviviruses, which include Dengue (DV) and West Nile (WN), mutate in response to immune system pressure. Identifying escape mutants, variant progeny that replicate in the presence of neutralizing antibodies, is a common way to identify functionally important residues of viral proteins. However, the mutations typically occur at variable positions on the viral surface that are not essential for viral replication. Methods are needed to determine the true targets of the neutralizing antibodies.

**Results:**

Stereophysicochemical variability plots (SVPs), 3-D images of protein structures colored according to variability, as determined by our PCPMer program, were used to visualize residues conserved in their physical chemical properties (PCPs) near escape mutant positions. The analysis showed 1) that escape mutations in the flavivirus envelope protein are variable residues by our criteria and 2) two escape mutants found at the same position in many flaviviruses sit above clusters of conserved residues from different regions of the linear sequence. Conservation patterns in T-cell epitopes in the NS3- protease suggest a similar mechanism of immune system evasion.

**Conclusion:**

The SVPs add another dimension to structurally defining the binding sites of neutralizing antibodies. They provide a useful aid for determining antigenically important regions and designing vaccines.

## Background

Flaviviruses, +-strand RNA viruses that cause diseases such as yellow fever (YF), Japanese encephalitis (JE), West Nile (WN), tick-borne encephalitis (TBE) and Dengue fever (DV), are endemic in many parts of the world. While some flaviviruses have relatively stable sequences, others are extremely variable. For example, some have suggested the term "quasispecies" for DV, as several different virus sequences could be isolated from the same blood sample [1,2]. The many asymptomatic human and animal carriers of these viruses represent an enormous reservoir for the development of new strains[3,4]. Continuous mutation at positions that are non-essential for replication allows flaviviruses to evade or confuse the immune system. This contributes to the development of fatal infections, such as Dengue hemorrhagic fever (DHF) [5,6]. To be effective, vaccines must induce efficient T-cell [7,8] and neutralizing antibody responses to functionally essential areas of the viral proteins[9].

Previous efforts to identify residues in flaviviruses that are essential for function have used escape mutants, viral progeny that survive in the presence of neutralizing antibodies to the virus [10-16]. However, while escape variants may have altered phenotypes[10,14], they do not prevent the replication of the virus, implying that the mutations are in residues not essential for function[17]. Here, we present a method that can be used to interpret escape mutations in a different way, by detecting conserved residues that are "cloaked" by these variable positions. These invariant residues are more likely to be the important targets of neutralizing antibodies the escape mutants, which typically occur at variable positions.

The method depends on our PCPMer program for analyzing variability, according to physicochemical properties of the amino acids, in sequence alignments. We have shown that the position specific variability data generated by the program, when coupled with structural analysis, can be used to identify areas that are important for function in families of related proteins [18-22]. Here, as a paradigm for the use of the method in vaccine design, we applied this method to the analysis of escape mutants of flaviviruses.

We used the PCPMer program to define areas conserved in physical chemical properties (PCP-motifs) of DV proteins of known structure. We then color coded the structures according to PCP-variability, and marked the position of known escape mutants and viral epitopes. The data divided the protein surface into a variable face, where all the escape mutants mapped, and a more conserved face. These areas were consistent with those previously defined by experimental methods [23-26]. We find that the escape mutants found in the same position in several different flaviviruses lie above highly conserved, known functional areas of the viral proteins, such as the receptor binding site, and disulfide bonded residues. These cloaked residues are more likely to be the true target for a neutralizing antibody.

## Results

### Defining PCP-motifs of DV proteins with PCPMer

A "PCP-motif" is an area in a group of related proteins with conserved physical chemical properties (PCPs). We have shown in previous work that PCP-motifs correspond to functional areas of proteins and can be used to identify functional homologues in sequence databases ([27,21]). The PCP motifs for two DV proteins of known structure, the Envelope and the serine protease domain of the non-structural protein NS3 are shown in Tables 1 and 2. For convenience in this paper, the motifs are given as areas of the Dengue virus protein sequence, rather than as the matrix of numbers relating to the conserved properties at each position that is their actual description (see methods).

**Table 1:** PCP-Motifs identified for the flavivirus Envelope proteins, using the sequence of DV-2env to indicate the sequence location and representative sequence. PCPMer parameters were: Gap cutoff of 2, length cutoff of 5 and the relative entropy range between 1 and 2.5 with a step of 0.1.

Motif No.	PCP-motifs
1	9 **R**DFVEGVSG 17*^i^
2	24 VLEHGSCVTTMAKNKPTLD 42
3	54 ATLRKYCIEA 63
4	74 CPTQGEP 80^F^
5	98 DRGWGNGCGLFGKGG 112^F^
6	116 CAMFTC 121
7	133 ENLEYTV 139
8	151 VGNDT 155
9	159 GKEVKITPQSS 169
10	175 LTGYGTVTMEC 185
11	197 VLLQMK 202
12	209 HRQWFLD 215
13	240 FKNP**H**AKKQDV 250^F^
14	281 GHLKCRLRMDKLQLKGMSYSMC 302
15	314 ETQHGT 319
16	332 PCKIPF 337
17	349 GRLITVNP 356
18	368 **E**AEPPFGD 375**
19	391 WFKKGSSIGQ 400
20	416 GDTAWDFGSLGG 427
21	431 SIGKALHQVFGAI 443
22	448 FSGVSW 453
23	459 IGVIITWIGMNSR 471
24	475 LSVSLVLVGVVTLYL 489

*residues 9–12 are at the C-terminal end of the CD8-T-cell epitope mapped for yellow fever [46]

**a Y to H mutation in TBE virus just after this motif is attenuating for neurovirulence [44]

^F ^Motifs that form the "fusion tip area" of domain II. Mutation of the analogous residue in TBE to the bold and underlined H in motif 13 prevents fusion [32].

^i ^Residues equivalent to those that form a salt bridge in the TBE-envelope are **bold **(R9/E368), those in the interface of the trimer of the TBE envelope protein are underlined [31].

**Table 2:** PCP-motifs identified for the flavivirus NS3 proteases, using the sequence of DV-2 NS3 to indicate location and representative sequence. The catalytic residues (H51, D75, S135) are shadowed; residues in the substrate interaction pocket [28] are **bold**, and areas that are part of known T-cell epitopes are underlined.

Motif No.	PCP-motifs
1	2 GVLWDVPSP 10
2	29 GILGYS**QI**GAG 39
3	43 EGTFHTMW**HV**TRGA 56
4	73 KK**D**LISYGGGW 83*
5	95 VQVLALEPG 103
6	133 **G**T**SG**SP 138**
7	148 GL**YGNG **153
8	159 G**AY**V**S**AIAQ 167

*C-terminal end, cytotoxic T-cell epitope AA 71–79 [40].

**N-terminal end of 11 mer T-cell activating peptide [5].

The PCP-motifs include all the known functional areas of the proteins, according to previous experimental results, and indicate areas that are most probably responsible for the activities that are common to all the flaviviruses. For example, the motifs of the NS3 protease (Table 2) include all the catalytic amino acids and all but one of the residues that interact with a peptide substrate analogue in a crystal structure of the complex[28].

### Mapping PCP-motifs of the DV-Env protein defines a conserved face and a fusion tip region

Mapping the motifs of Table 1 on the 3D structure, determined by X-ray crystallography, of the DV-2 envelope protein (DV residues 281– 674)[29,30](Figure 2a) shows that they map primarily to one, conserved face of the molecule. Many of these sequences are involved in interdomain and trimer interactions of the envelope protein from TBE[31]. The plot reveals that three of the motifs occur near one another at the end of Domain II. These are the previously defined "fusion peptide" and two other loops that are as much as 140 amino acids away in the linear sequence of the protein (the three areas are marked by ^F ^in Table 1). This suggests that the whole tip of the protein is involved in fusion. We note that mutation in one of these loops in TBE (at the absolutely conserved H shaded in Table 1) does indeed effect viral fusion[32].

**Figure 1:**
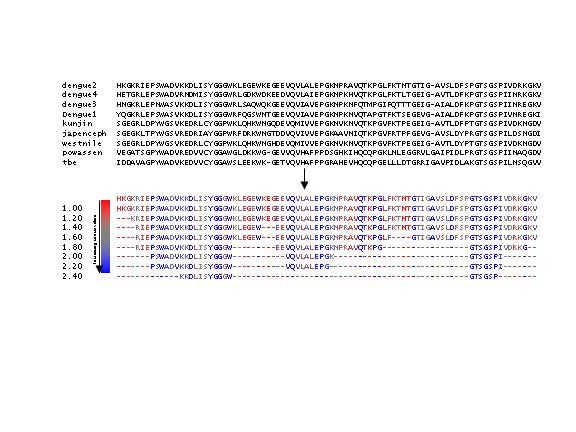
**The relative specific entropy (SE) function of PCPMer (Bin Zhou et al., in preparation) defines motifs even in alignments where the sequence conservation varies locally. **The top of the figure shows a section of the sequence alignment for the NS3 protein. The next section shows the PCPMer output, indicating the motifs in the NS3 protease according to the sequence of DV-2 as a function of the specific entropy level (numbers to the left). PCPMer parameters were: Gap cutoff of 2, length cutoff of 5, relative entropy range between 1 and 2.5 with a step of 0.2. Note the conserved sequences around the active site residues (bold letters) of the protease are followed by variable regions that retain conservation in one of the five physical chemical property vectors. The output is colored to reflect the degree of conservation at each position.

**Figure 2:**
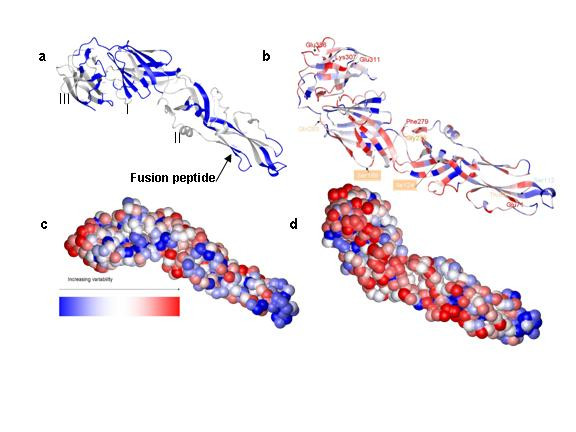
**Variability analysis of the envelope protein of DV-2 and illustration of how escape mutants mark cloaked conserved residues. a) **PCP-motifs (blue) common to all flavivirus envelope proteins are mapped on the structure of DV-2 Env (PDB file 1OAN; the start and end residues are numbered). Note the high conservation of the fusion peptide (arrow) and two loop regions adjacent to it from other areas of the molecule. **b) **Stereochemical variability plot (SVP) of the DV2-Env (PDB file 1OKE), showing the per residue variation across the Flaviriridiae. Known escape mutants of DV-2 and DV-3 [10, 26] are labeled and the residue names are colored according to their variability. The boxed residues are intermediate in the conservation scale (white). **c **and **d**) Surface plots of the SVP shown in figure 2B, showing the conserved (overall blue, **c**) face, where the motifs of conserved areas map. The variable face (**d**, mostly red), which matches the orientation of the molecule where the escape mutants map to.

### Visualizing clusters of conserved residues from different sequence areas with SVPs

**Figure 3:**
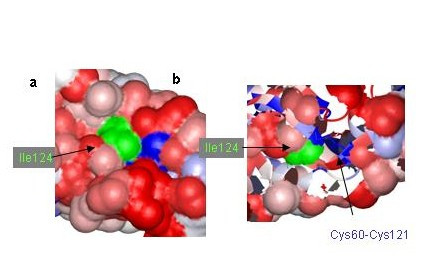
**a) **Local surface plot of the DV-Env SVP around residue 124 (which is highly variable but has been colored green here for clarity), illustrating how the residue forms part of a patch of variable (red) residues **b**) Removing part of the surface reveals how I124 lies above the highly conserved residues Cys60-121 (disulfide bonded) and Tyr59 that are distant in the sequence of the protein.

**Figure 4:**
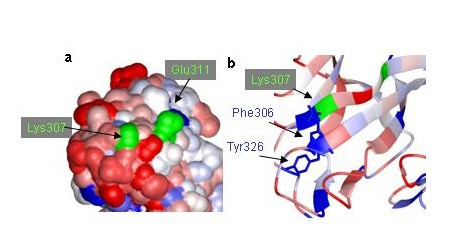
**a) Local surface plot of the area around two escape mutant positions in domain III. **Residues 307 and 311 are highly variable and have been colored green here for visibility. The faint blue area on the surface near residue 307 comes from a highly conserved aromatic residue, Phe306, which lies under the variable residue and forms a cluster with another conserved residue, Tyr326 (b).

**Figure 5:**
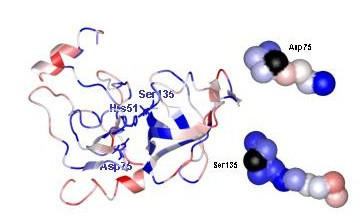
**The conserved essential residues in two serotype specific T-cell epitopes of the NS3 protease are followed by variable residues that will affect MHC binding. **The crystal structure of the NS3 protease of DV-2 is colored according to sequence variability across the flaviviruses(see figure 1 and 2 for details). The catalytic triad sidechains are shown in neon and labeled. The T-cell epitopes (right) around the catalytic residues Asp75 (residues 71–79), and Ser135 (133–143), are shown as space filling and color coded to reflect variability, except that the 100% conserved catalytic residues are both black.

Alternatively, the conservation of each amino acid, represented by the specific entropy, [33,34], as described in more detail in Figure 1 and Methods, can be mapped onto a protein's 3-D structure, by coloring each amino acid. The higher the specific entropy, the more conserved the position. These 3-D plots, which we refer to as "stereophysicochemical variability plots" (Figure 2, 3, 4, 5) can be used to find conserved areas of the protein, distant in the sequence, that are close together in 3D space.

The SVP can be used to define the amino acid profile of antibody binding sites, which have been localized by peptide mapping or escape mutants. An SVP for the DV-env structure (Figure 2b–d), with the specific entropy of each residue shown by color, shows the two faces, one conserved (where the PCP-motifs map; 2c) and one variable (2b). This is also in accord with another analysis of conservation in this protein, where only the identical residues in these 14 flaviviruses were plotted[35]. The SVP indicates that most of the residues on the "inner" face of the envelope protein (Figure 2c) are conserved in at least one PCP-vector, even if the sequence at these positions varies.

### Escape mutants of the DV-Env protein are in variable positions near conserved residues

Known escape mutants of DV[10,26] map to the variable face of the SVP (Figure 2b, 2d) and are generally in highly variable positions. This is consistent with a previous sequence analysis of mutants of tick-borne flaviviruses [36]. Those in the more conserved positions show a limited range of alteration in the progeny escape virus. For example, Residue 112, which is at the border of the fusion peptide motif, is the most conserved of the mutants. This residue is either an S or G in all the flavivirus sequences, with one escape mutant described as "S112G"[26].

### Lifting the cloak: the hidden essential residues

To illustrate how the method can be used to define the target of neutralizing antibodies, the areas around two escape mutants in the Env protein of DV, both in positions where escape mutations occur in many different flaviviruses, were analyzed.

#### Example 1: Residue 124 in domain 1

Type specific neutralizing antibodies that bind near this position have been found for four other flaviviruses[26], suggesting that the area cloaked by this residue constitutes an area essential for function. Surviving progeny with mutations at this position either conserve the residues hydrophobicity (YF-17D: Met125Ile), or convert it to a hydrophilic residue (DV: I124N, JE: I126T, MVE: A126E, TBE: A123K).

Zooming in on this region (figure 3a, detail) reveals that Ile124 lies above the PCP- motif 54–63 in the folded structure. The side chains of residues around it cloak two cysteines that are disulfide bonded, Cys60 and Cys121, and the highly conserved residue Tyr 59 (figure 3b). This suggests that converting Ile124 and the residues that surround it in the 3D-structure to small hydrophilic residues should enhance the immunogenicity of the areas below that must be blocked to obtain neutralization by the antibodies.

#### Example 2: Residue 307 in domain 3

Two DV escape mutants positions, 307 and 311, are part of a variable surface in domain III (figure 4a). A mutation at position 307 has also been observed in other flaviviruses. For example, a Lys307Glu mutation has been implicated in attenuating West Nile neurovirulence[13]. Further, this mutation and another that is close to it structurally, at position 330, block the binding of several antibodies that neutralize WN replication[17]. Attenuated Tick borne encephalitis virus was obtained by mutating residues near this position, which is considered to be an important site for receptor binding[37]. All this indicates that while residue 307 can vary, some residue near it must be essential and conserved.

According to our analysis, Residue 307 and the variable residues near it cloak two highly conserved aromatic residues, Phe 306 and Tyr 326, that overlap each other (Figure 4b). Similar analysis (not shown) of the NMR structure of WN-env in this area points to the equivalent residues being cloaked by two escape mutants (at positions 307 and 330 in WN), which both block neutralization by three different monoclonal antibodies[17]. We suggest that these aromatic residues contribute to the epitopes detected by the antibodies, and that the antibody prevents wild type virus replication by blocking their conformational change during receptor binding. In our alignment of the Flaviviruses (supplementary data), only the 17D vaccine strain of Yellow fever varies at these positions. Mutation studies are now underway to determine whether these residues play a role in attenuation.

### T-cell epitopes in NS3 protease: variable residues alter binding to peptides containing essential conserved positions

A similar pattern can be seen with T-cell epitopes, which are also important determinants of the immune response [38,39]. Two dominant T-cell epitopes have been identified in the NS3 of DV, the protease that cleaves the polyprotein at several positions, at residues 71–79[40] and 133–143[5]. Both of these epitopes contain amino acids that are essential for protease activity, D75 and S135 respectively. In the SVP representation of the crystal structure [41] of the DV-2 NS3 protease domain (Figure 5), both the conserved essential amino acids are surrounded by variable residues which will alter binding to T-cell antigens. This could be the basis for reduced binding to the immune surveillance (HLA alleles) and clearance mechanisms in infected individuals. For the first epitope, the determinant for cytotoxic T-cell activation by DV-2 or DV-3 serotype is in one variable amino acid, D71[40], which is S71 in DV-3 serotypes (Figure 1). This sort of analysis can aid in choosing vaccine strains, on the basis of how their sequences conform to the known binding signatures for major MHC alleles, while retaining viral replication in cell culture.

## Discussion

While residue conservation has long been recognized as a way to detect important areas of viral proteins[35,42,43], the new tools presented here for distinguishing conservation with respect to the PCPs of the amino acid side chains provide a rapid way to interpret an ensemble of sequence and escape mutant data. Plotting per residue conservation of physical chemical properties in the form of SVPs permits one to rapidly detect which residues are most likely to contribute to the epitope face (Figure 2b,d), and which are most important for the function of the virus (Figure 2a,c, Table 1 and 2). As we show, the mathematical methods are robust to sample size (in this case, PCPmer analysis with 8 or 14 dependable virus sequences gave similar plots, as described in Methods), and the specific entropy criterion is a useful measure of the importance of residues. For example, the PCP-motifs in the NS3 protease domain contain all amino acids known to be important for function and substrate binding (Table 2 and Figure 5).

Applying the PCPMer decomposition methods to the flavivirus family aided in interpreting experimental results and suggested site specific alterations that can be tested for vaccine design. PCPMer motifs, combined with structural analysis, are a rapid way to identify functionally important areas of proteins[21,22]. Colored SVPs supply a fourth dimension, variability, to a crystal or model structure, that is a valuable aid in interpreting experimental data. Mapping known escape mutants on SVPs shows they occur in areas of high variability. While the altered residues in escape mutants may have deleterious effects under some growth conditions[10,14,36,44], they are confined to residues that are not essential for replication. The masked, conserved residues below them are more likely to be important. For example, the escape mutants at positions 124 and 307 (Figure 3 and 4) mark the site of antibody binding, but the SVP suggests that conserved amino acids, close in space but 20–60 residues away in the linear sequence, are more important for neutralization. Similar principles may also apply to T-cell epitopes, according to the NS3 example (Figure 5). The SVP can further be used to suggest other amino acids in a composite site could be altered to better direct antibodies to an essential area of the protein.

An effective immune response must be generated against the conserved regions of the viral proteins, and not be diverted by the variable cloak around them. Our analysis of the whole range of flaviviruses indicates that physicochemical properties remain constant even in stretches of residues that would appear by other measures to be variable. The SVP methodology provides a novel way to select mutants or design a virus so as to enhance the accessibility of the conserved residues that are normally cloaked.

## Conclusion

We have shown that PCP motifs and SVPs provide a rapid method to obtain information from viral sequence data. Once identified, these areas can be used to design vaccine candidate recombinant viruses or individual proteins that will more efficiently stimulate an effective immune response to these essential areas. We anticipate that the PCPmer program and related visualization tools will be a routine method in the future for analyzing sequence data of variable virus sequences.

## Methods

### Variability analysis

A large set (237) of physicochemical properties of amino acid side chains were reduced to five descriptors (E1-E5) by multidimensional scaling [34]. The 5 descriptors summarize all known quantitative properties that differentiate the sidechains, including among others the hydrophobicity (defined in numerous ways), amino acid size, tendency to occur in secondary structures, charge, binding to various affinity chromatography columns. These descriptors offer an alternative to the commonly used scoring matrices, such as the PAM series and Gonnet, which are based on statistical analysis of amino acid substitutions, to determine areas of residue conservation in proteins. Our program suite, PCPMer , defines areas of conservation in aligned protein sequences according to the values of the five vectors at each position in a sequence alignment [27,34]. The user specified values dictate allowed gaps, minimum length, and entropy range for the motifs. To determine the information content of the pattern of residue properties in a column of the multiple alignment, MOTIFMAKER determines a "specific entropy" value of the component *E*^*i *^at position *k *relative to the expected random distribution [27] :



The term "entropy" is used here as it is in information science, as a measure of the uncertainty of a given event [45]. The relative entropy is thus the observed conservation of the physical chemical property vectors (b = 1–5) of residues in a column relative to that which would be expected if the position varied randomly. In this case, a high specific entropy indicates that the conservation in a column of a multiple alignment, according to a given physical property vector, is significantly greater than chance.

### Relative specific entropy

Even in variable areas of sequence alignments, there may be a pattern of conservation in one of the vectors that underlies the amino acid sequence diversity. A relative specific entropy scale can be used to determine motifs in alignments where the variability depends on position in the sequence. Alternatively, a sliding entropy scale can be used to define particularly conserved regions in alignments that are generally more homogeneous in character. Figure 1 illustrates the usefulness of this feature. The top rows show a section of the alignment of NS3 proteins from 8 flaviviruses. The lower section shows the output of PCPMer for this area, according to which residues in the top (marker) sequence would be part of a motif at each specific entropy level. The program automatically takes the most highly conserved areas in each section of the alignment to be motifs.

### PCP-motif definition

The user can choose the minimum length of motifs ("length cutoff"), the maximum number of variable positions between two conserved ones ("Gap cutoff") and the specific entropy range for defining motifs in PCPMer. For the sake of simplicity in this paper, the motifs in Figure 1 and Tables 1 and 2 are given according to the top sequence in the alignment from which they were derived. However, the actual definition of the PCP-motifs is a series of numerical matrices, that define the type and degree of conservation of the physical chemical properties of each column in the original sequence alignment. These matrices can be used to automatically scan sequence databases, using the MOTIFMINER program, to identify proteins that contain sequences similar to the PCP-motifs defined for the initial set of proteins [27]. In this work, we have chosen to identify PCP-motifs as highly conserved areas of the viral proteins whose conservation would indicate an important functional or structural role.

### Stereophysicochemical variability plots

The plots of Figures 2 and 3 were drawn with MOLMOL, from the PDB coordinate files of the crystal structures, using a macro that colors each residue position according to the specific entropy values determined by PCPMer. The median of a histogram of the number of residues at each specific entropy level was defined as the midpoint in the color scheme. The highest specific entropy for any residue was set to blue and the lowest to red.

### Flavivirus alignment

The whole genome sequences of 8 flaviviruses that included representatives of each DV serotype were downloaded from GENBANK and the areas for the envelope and NS3 protein selected (alignments are provided as supplementary data). The sequences were aligned with CLUSTALW using a GONNET matrix and standard set conditions. This alignment was used for the initial analysis of the envelope protein and the analysis of the NS3 protease shown in Figures 1 and 3. To test the robustness of the method, a second alignment of 14 flavivirus sequences (Supplementary data 1; used for Figures 2, 3, 4) for the envelope protein was generated and the analysis was repeated. There was little or no difference in the positional variability or the specific entropy calculations with the larger number of sequences, but the ends of two of the motifs were slightly different.

## Competing interests

The author(s) declare that they have no competing interests.

## Authors' contributions

**Catherine H. Schein**: Sequence decomposition, interpretation of results; alignment design and data analysis

**Bin Zhou**: chief programmer of the PCPMer program, prepared SVPs, sequence analysis

**Werner Braun**: design of the PCPMer method

## Supplementary Material

Additional File 1 **Multiple sequence alignment (CLUSTAL W (1.82)) of 14 flavirus envelope sequences**. Escape mutant positions are bold, the conserved residues in domains I (Y59, C60, C121) and III (F306, Y326) that are near escape mutants common to several flaviviruses are in red. Corresponding residues that are variant in the yellow fever 17D strain are in bold and underlined. Click here for file
