# Supplementary material for: Stereophysicochemical variability plots highlight conserved antigenic areas in Flaviviruses
Source: Virol J. 2005 Apr 21;2:40. doi: 10.1186/1743-422X-2-40 (PMC1112618; doi:10.1186/1743-422X-2-40)
Supplement: Additional File 1 — Multiple sequence alignment (CLUSTAL W (1.82)) of 14 flavirus envelope sequences. Escape mutant positions are bold, the conserved residues in domains I (Y59, C60, C121) and III (F306, Y326) that are near escape mutants common to several flaviviruses are in red. Corresponding residues that are variant in the yellow fever 17D strain are in bold and underlined. [file 1743-422X-2-40-S1.doc]

**Schein et al. supplementary data 1.**

CLUSTAL W (1.82) multiple sequence alignment of 14 flavirus envelope sequences. Escape mutant positions are bold, the conserved residues in domains I (Y59,C60, C121) and III (F306, Y326)that are near escape mutants common to several flaviviruses are in red. Corresponding residues that are variant in the yellow fever 17D strain are in bold and underlined.

1OANdengue2 MRCIGISNRDFVEGVSGGSWVDIVLEHGSCVTTMAKNKPTLDFELIKTEAKQPATLRKYC 60

Dengue4 MRCVGVGNRDFVEGVSGGAWVDLVLEHGGCVTTMAQGKPTLDFELTKTTAKEVALLRTYC 60

POLG_DEN3 MRCVGVGNRDFVEGLSGATWVDVVLEHGGCVTTMAKNKPTLDIELQKTEATQLATLRKLC 60

Dengue1 MRCVGIGNRDFVEGLSGATWVDVVLEHGSCVTTMAKDKPTLDIELLKTEVTNPAVLRKLC 60

STEVM FNCLGTSNRDFVEGASGATWIDLVLEGGSCVTVMAPEKPTLDFKVMKMEATELATVRKYC 60

westnileDB FNCLGMSNRDFLEGVSGATWVDLVLEGDSCVTIMSKDKPTIDVKMMNMEAANLAEVRSYC 60

JapEVenv FNCLGMGNRDFIEGASGATWVDLVLEGDSCLTIMANDKPTLDVRMINIEAS**Q**LAEVRSYC 60

POLG_MVEV FNCLGMSSRDFIEGASGATWVDLVLEGDSCITIMAADKPTLDIRMMNIEATNLALVRNYC 60

KUNJM FNCLGMSNRDFLEGVSGATWVDLVLEGDSCVTIMSKDKPTIDVKMMNMEAANLAEVRSYC 60

YEFV_17D AHCIGITDRDFIEGVHGGTWVSATLEQDKCVTVMAPDKPSLDISLETVAIDRPAEVRK**V**C 60

1SVKtbe SRCTHLEN**R**DFVTGTQGTTRVTLVLELGGCVTITAEGKPSMDVWLDAIYQENPAKTREYC 60

POLG_POWVL TRCTHLENRDFVTGTQGTTRVSLVLELGGCVTITAEGKPSIDVWLEDIFQESPAETREYC 60

Langat SRCTHLENRDFVTGVQGTTRLTLVLELGGCVTVTADGKPSLDVWLDSIYQESPAQTREYC 60

Loupill SRCTHLENRDFVTGTQGTTRVTLVLELGGCVTITAEGKPSMDVWLDAIYQESPAKTREYC 60

.* .***: * * : : .** . *:* : **::*. : * * *

R9

1OANdengue2 IEAKLTNT**T**T**E**SRCPTQGEPTLNEEQDKRFVCKHSMVDRGWGNGCGLFGKGGIVTCAMFT 120

Dengue4 IEASISNITTATRCPTQGEPYLKEEQDQQYICRRDVVDRGWGNGCGLFGKGGVVTCAKFS 120

POLG_DEN3 IEGKITNITTDSRCPTQGEAILPEEQDQNYVCKHTYVDRGWGNGCGLFGKG**S**LVTCAKFQ 120

Dengue1 IEAKISNTTTDSRCPTQGEATLVEEQDANFVCRRTFVDRGWGNGCGLFGKGSLITCAKFK 120

STEVM YEATLDTLSTVARCPTTGEAHNTKRSDPTFVCKRDVVDRGWGNGCGLFGKGSIDTCAKFT 120

westnileDB YLATVSDLSTKAACPTMGEAHNDKRADPAFVCRQGVVDRGWGNGCGLFGKGSIDTCAKFA 120

JapEVenv YHASVTDISTVARCPTTGEAHNEKRADSSYVCKQGFTDRGWGNGCGLFGKGSIDTCAKFS 120

POLG_MVEV YAATVSDVSTVSNCPTTGESHNTKRADHNYLCKRGVTDRGWGNGCGLFGKGSIDTCAKFT 120

KUNJM YLATVSELSTKAACPTMGEAHNDKRADPSFVCKQGVVDRGWGNGCGLFGKGSIDTCAKFA 120

YEFV_17D YNAVLTHVKI**ND**KCPSTGEAHLAEENEGDNACKRTYSDRGWGNGCGLFGKGSIVACAKFT 120

1SVKtbe LHAKLSDTKV**A**ARCPTMGPATLAEEHQGGTVCKRDQSDRGWGNHCGLFGKGSIVACVKAA 120

POLG_POWVL LHAKLTNTKVEARCPTTGPATLPEEHQANMVCKRDQSDRGWGNHCGFFGKGSIVACAKFE 120

Langat LHAKLTGTKVAARCPTMGPATLPEEHQSGTVCKRDQSDRGWGNHCGLFGKGSIVTCVKFT 120

Loupill LHAKLSETKVAARCPTMGPAVLTEERQIGTVCKRDQSDRGWGNHCGLFGKGSIVACVKAA 120

. : . **: * . :. : *:: ****** **:****.: :*.

"Fusion peptide"

1OANdengue2 CK--KNMEGKIVQPENLEYTVVITPH-SGEEHAVGNDTGKHGKEVK----ITPQSSITEA 173

Dengue4 CS--GKITGNLVRIENLEYTVVVTVH-NGDTHAVGNDTSNHGVTAM----ITPRSPSVEV 173

POLG_DEN3 CL--ES**I**EGKVVQHENLKYTVIITVH-TGDQHQVGNET--QGVTAE----ITSQASTAEA 171

Dengue1 CV--TKLEGKIVQYENLKYSVIVTVH-TGDQHQVGNESTEHGTTAT----ITPQAPTSEI 173

STEVM CK--NKATGKTILRENIKYEVAIFVHGSTDSTSHGNYSEQIGKNQAARFTISPQAPSFTA 178

westnileDB CS--TKAIGRTILKENIKYEVAIFVHGPTTVESHGNYSTQVGATQAGRFSITPAAPSYTL 178

JapEVenv CT--SKA**I**GRTIQPENI**K**YEVGIFVHGTTTSENHGNYSAQVGASQAAKFTVTPNAPSITL 178

POLG_MVEV CS--NSA**A**G**R**LILPEDIKYEVGVFVHGSTDSTSHGNYSTQIGANQAVRFTISPNAPAITA 178

KUNJM CS--TKATGRTILKENIKYEVAIFVHGPTTVESHGNYFTQTGAAQAGRFSITPAAPSYTL 178

YEFV_17D CA--KS**M**SLFEVDQTKIQYVIRAQLHVGAKQENWNT**D**IK**T**LKFDAL--------SGSQEV 170

1SVKtbe CE**A**KKKATGHVYDANKIVYTVKVEP**H**--TGDYVAANETHSGRKTAS----FTISSE**K**TIL 174

POLG_POWVL CEEAKKAVGHVYDSTKITYVVKVEPH--TGDYLAANETNSNRKSAQ----FTVASEKVIL 174

Langat CEDKKKATGHVYDVNKITYTIKVEPH--TGEFVAANETHSGRKSAS----FTVSSEKTIL 174

Loupill CEAKKKATGYVYDANKIVYTVKVEPH--TGDYVAANETHKGRKTAT----FTVSSEKTIL 174

* . .: * : * . :

H146

1OANdengue2 ELTGYGTVTMECSPRTGLDFNEMVLLQMKDK------AWLVHRQWFLDLPLPWLPGADTQ 227

Dengue4 KLPDYGELTLDCEPGLELYFNEMILMKMKKK------TWLVHKQWFLNLPLPWTAGADTS 227

POLG_DEN3 ILPEYGTLGLECSPRTGLDFNEMILLTMKNK------AWMVHRQWFFDLPLPWTSGATTK 225

Dengue1 QLTDYGALTLDCSPRTGLDFNEMVLLTMKEK------SWLVHKQWFLDLPLPWTSGATTS 227

STEVM NMGEYGTVTIDCEARSGINTEDYYVFTVKEK------SWLVNRDWFHDLNLPWTSPATT- 231

westnileDB KLGEYGEVTVDCEPRSGIDTNAYYVMTVGTK------TFLVHREWFMDLNLPWSSAGST- 231

JapEVenv KLGDYGEVTLDCEPRSGLNTEAFYVMTVGSK------SFLVHREWFHDLALPWTSPSST- 231

POLG_MVEV KMGDYGEVTVECEPRSGLNTEAYYVMTIGTK------HFLVHREWFNDLLLPWTSPAST- 231

KUNJM KLGEYGEVTVDCEPRSGIDTSAYYVMTVGTK------TFLVHREWFMDLNLPWSSAESN- 231

YEFV_17D EFIGYGKATLECQVQTAVDFGNSYIAEMETE------SWIVDRQWAQDLTLPWQSGSGG- 223

1SVKtbe TMGEYG**D**VSLLCRVASGVDLAQTVILELDKTVEHLPTAWQVHRDWFNDLALPWKHEGA**Q**- 233

POLG_POWVL RLGDYGDVSLTCKVASGIDVAQTVVMSLDSSKDHLPSAWQVHRDWFEDLALPWKHKDNQ- 233

Langat TLGDYGDVSLLCRVASGVDLAQTVVLALDKTHEHLPTAWQVHRDWFNDLALPWKHDGAE- 233

Loupill TLGEYGDVSLLCRVASGVDLAQTIILELDKTAEHLPTAWQVHRDWFNDLALPWKHDGNP- 233

: ** : * : : : : *.::* :* ***

1OANdengue2 GSNWIQKETLVTFKNPHAKKQDVVVLGSQEGAMHTALTGATEIQMSSG----NLL**F**TGHL 283

Dengue4 EVHWNYKERMVTFKVPHAKRQDVTVLESQEGAMHSALAGATEVDSGDG----NHMFAGHL 283

POLG_DEN3 TPTWNRKELLVTFKNAHAKKQEVVVLGSQEGAMHTALTGATEIQTSGG----TSIFAGHL 281

Dengue1 QETWNRQDLLVTFKTAHAKKQEVVVLGSQEGAMHTALTGATEIHTSGT----TTIFAGHL 283

STEVM --DWRNRETLVEFEEPHATKQTVVALGSQEGALHTALAGAIPATVSSST---LTLQSGHL 286

westnileDB --VWRNRETLMEFEEPHATKQSVIALGSQEGALHQALAGAIPVEFSSNT---VKLTSGHL 286

JapEVenv --AWRNRELLMEFEEAHATKQSVVALGSQEGGLHQALAGA**I**VVEYS**S**S----VKLTSGHL 285

POLG_MVEV --EWRNREILVEFEEPHATKQSVVALGSQEGALHQALAGAIPVEFSSST---LKLTSGHL 286

KUNJM --VWRNRETLMEFEEPHATKQSVIALGSQEGALHQALAGAIPVEFSSNT---VKLTSGHL 286

YEFV_17D --VWREMHHLVEFEPPHAATIRVLALGNQEGSLKTALTGAMRVTKDTNDNNLYKLHGGHV 281

1SVKtbe --NWNNAERLVEFGAPHAVKMDVYNLGDQTGVLLKALAGVPVAHIEGTK---YHLKSGHV 288

POLG_POWVL --DWNSVEKLVEFGPPHAVKMDVFNLGDQTAVLLKSLAGVPLASVEGQK---YHLKSGHV 288

Langat --AWNEAGRLVEFGTPHAVKMDVFNLGDQTGVLLKSLAGVPVASIEGTK---YHLKSGHV 288

Loupill --HWNNAERLVEFGAPHAVKMDVYNLGDQTGVLLRALAGVPVAHIEGNK---YHLKSGHV 288

* :: * .** * * .* . : :*:*. : **:

1OANdengue2 KCRLRMDKLQLKGMSYSMCTG-KF**K**VVK**E**IAETQHGTIVIRVQYEGDGSPCKIPFEIMD- 341

Dengue4 KCKVRMEKLRIKGMSYTMCSG-KFSIDKEMAETQHGTTVVKVKYEGAGAPCKVPIEIRD- 341

POLG_DEN3 KCRLKMDKLKLKGMSYAMCLN-TFVLKKEVSETQHGTILIKVEYKGEDAPCKIPFSTED- 339

Dengue1 KCTLKMDKL**T**LKGMSYVMCTG-SF**K**LEKEVAETQHGTVLVQVKYEGTDAPCKIPFSTQD- 341

STEVM KCRAKLDKVKIKGTTYGMCDS-AFTFSKNPTDTGHGTVIVELQYTGSNGPCRVPISVTAN 345

westnileDB KCRVKMEKLQLKGTTYGVCSK-AFKFLGTPADTGHGTVVLELQYTGTDGPCKVPISSVAS 345

JapEVenv KCRLKMDKLALKGTTYGMCTG-KFSFAKNPADTGHGTVVIELSYSGRD**G**PCKIPIVSVAS 344

POLG_MVEV KCRVKMEKLKLKGTTYGMCTE-KFTFSKNPADTGHGTVVLELQYTGSDGPCKIPISSVAS 345

KUNJM KCRVKMEKLQLKGTTYGVCSK-AFRFLGTPADTGHGTVVLELQYTGTDGPCKIPISSVAS 345

YEFV_17D SCRVKLSALTLKGTSYKICTD-K**M**FFVKNPTDTGHGTVVMQVK**V**S-KGAPCRIPVIVADD 339

1SVKtbe TCEVGLEKLKMKGLTYTMCDKTKFTWKRAPTDSG**H**DTVVMEVTFSGT-KPCRIPVRAVAH 347

POLG_POWVL TCDVGLEKLKLKGTTYSMCDKAKFKWKRVPVDSGHDTVVMEVSYTGSDKPCRIPVRAVAH 348

Langat TCEVGLEKLKMKGLTYTVCDKTKFTWKRAPTDSGHDTVVMEVGFSGT-RPCRIPVRAVAH 347

Loupill TCEVGLEKLKMKGLTYTMC**D**K**SK**FAWKRTPTDSGHDTVVMEVTFSGS-KPCRIPVRAVAH 347

.* :. : :** :* :* : :: *.* ::.: **::*.

(H323)

1OANdengue2 LEKRHVLGRLITVNPIVTEK--DSPVNIEAEPPFGDSYIIIGVEPGQLKLNWFKKGSSIG 399

Dengue4 VNKEKVVGRIISSTPLAENT--NSVTNIELERPL-DSYIVIGVGNSALTLHWFRKGSSIG 398

POLG_DEN3 GQGKAHNGRLITANPVVTKK--EEPVNIEAEPPFGESNIVIGIGDKALKINWYRKGSSIG 397

Dengue1 EKGVIQNGRVITAHPIVTDK--EKPVHIEAEPPFGESYIVVGAGEKALKLSWFKKGSTIG 399

STEVM LMDLTPVGRLVTVNPFISTGGANNKVMIEVEPPFGDSYIVVGRGTTQINYHWHKEGSSIG 405

westnileDB LNDLTPVGRLVTVNPFVSVATANAKVLIELEPPFGDSYIVVGRGEQQINHHWHKSGSSIG 405

JapEVenv LNDMTPVGRLVTVNPFVATSSANSKVLVEMEPPFGDSYIVVGRGDKQINHHWHKAGSTLG 404

POLG_MVEV LNDMTPVGRMVTANPYVASSTANAKVLVEIEPPFGDSYIVVGRGDKQINHHWHKEGSSIG 405

KUNJM LNDLTPVGRLVTVNPFVSVSTANAKVLIELEPPFGDSYIVVGRGEQQINHHWHKSGSSIG 405

YEFV_17D LTAAINKGILVTVNPIASTN--DDEVLIEVNPPFGDSYIIVGRGDSRLTYQWHKEGSSIG 397

1SVKtbe GSPDVNVAML**I**TPNPTIENN---GGGFI**E**MQLPPGDNIIYVG----ELSHQWFQKGSSIG 400

POLG_POWVL GVPAVNVAMLITPNPTIETN---GGGFIEMQLPPGDNIIYVG----DLSQQWFQKGSTIG 401

Langat GVPEVNVAMLITPNPTMENN---GGGFIEMQLPPGDNIIYVG----DLNHQWFQKGSSIG 400

Loupill GSPDVNVAMLITPNPTIEND---GGGFIEMQLPPGDNIIYVG----ELSHQWFQTGSSIG 400

. ::: * :* : * :. * :* :. *.: **::*

**E373** Trimerint

1OANdengue2 QMFETTMRGAKRMAILGDTAWDFGSLGGVFTSIGKALHQVFGAIYGAAFSGVSWTMKILI 459

Dengue4 KMFESTYRGAKRMAILGETAWDFGSVGGLFTSLGKAVHQVFGSVYTTMFGGVSWMIRILI 458

POLG_DEN3 KMFEATARGARRMAILGDTAWDFGSVGGVLNSLGKMVHQIFGSAYTALFSGVSWIMKIGI 457

Dengue1 KMFEATARGARRMAILGDTAWDFGSIGGVFTSVGKLVHQIFGTAYGVLFSGVSWTMKIGI 459

STEVM KALATTWKGAQRLAVLGDTAWDFGSIGGVFNSIGKAVHQVFGGAFRTLFGGMSWITQGLL 465

westnileDB KAFTTTLKGAQRLAALGDTAWDFGSVGGVFTSVGKAVHQVFGGAFRSLFGGMSWITQGLL 465

JapEVenv KAFSTTLKGAQRLAALGDTAWDFGSIGGVFNSIGKAVHQVFGGAFRTLFGGMSWITQGLM 464

POLG_MVEV KAFSTTLKGAQRLAALGDTAWDFGSVGGVFNSIGKAVHQVFGGAFRTLFGGMSWISQGLL 465

KUNJM KAFTATLKGAQRLAALGDTAWDFGSVGGVFTSVGKAVHQVFGGAFRSLFGGMSWITQGLL 465

YEFV_17D KLFTQTMKGVERLAVMGDTAWDFSSAGGFFTSVGKGIHTVFGSAFQGLFGGLNWITKVIM 457

1SVKtbe RVFQKTRKGIERLTVIGEHAWDFGSTGGFLTSVGKALHTVLGGAFNSLFGGVGFLPKILV 460

POLG_POWVL RMFEKTRRGLERLSVVGEHAWDFGSVGGVLSSVGKAIHTVLGGAFNTLFGGVGFIPKMLL 461

Langat RVLQKTRKGIERLTVLGEHAWDFGSVGGVMTSIGRAMHTVLGGAFNTLLGGVGFLPKILL 460

Loupill RVFQTTRKGIERLTVIGEHAWDFGSAGGFFGSIGKAVHTVLGGAFNSIFGGVGFLPKLLM 460

: : * :* .*:: :*: ****.* **.: *:*: :* ::* : :.*:.: : :

1OANdengue2 GVIITWIGMNSRSTSLSVSLVLVGVVTLYLGAMVQA- 495

Dengue4 GFLVLWIGTNSRNTSMAMTCIAVGGITLFLGFTVQA- 494

POLG_DEN3 GVLLTWIGLNSKNTSMSFSCIAIGIITLYLGVVVQA- 493

Dengue1 GVLLTWLGLNSRSTSLSMTCIAVGLVTLYLGVMVQA- 495

STEVM GALLLWMGLQARDRSISLTLLAVGGILIFLATSVQA- 501

westnileDB GALLLWMGINARDRSIALTFLAVGGVLLFLSVNVHA- 501

JapEVenv GALLLWMGVNARDRSIALAFLATGGVLVFLATNVHA- 500

POLG_MVEV GALLLWMGVNARDKSIALAFLATGGVLLFLATNVHA- 501

KUNJM GALLLWMGINARDRSIALTFLAVGGVLLFLSVNVHA- 501

YEFV_17D GAVLIWVGINTRNMTMSMSMILVGVIMMFLSLGVGA- 493

1SVKtbe GVVLAWLGLNMRNPTMSMSFLLAGGLVLAMTLGVGA- 496

POLG_POWVL GVALVWLGLNARNPTMSMTFLAVGALTLMMTMGVGAD 498

Langat GVAMAWLGLNMRNPTLSMGFLLSGGLVLAMTLGVGA- 496

Loupill GVALAWLGLNTRNPTMSMSFLLAGGLVLAMTLGVGA- 496

* : *:* : :. :::. : * : : : * *
